# Ancel Keys, the Mediterranean Diet, and the Seven Countries Study: A Review

**DOI:** 10.3390/jcdd12040141

**Published:** 2025-04-08

**Authors:** Alessandro Menotti, Paolo Emilio Puddu

**Affiliations:** 1 Association for Cardiac Research, 00182 Rome, Italy; amenotti2@gmail.com; 2 EA 4650, Signalisation, Électrophysiologie et Imagerie des Lésions D’ischémie Reperfusion Myocardique, Normandie Université, 14000 Caen, France

**Keywords:** Mediterranean Diet, Seven Countries Study, coronary heart disease

## Abstract

The identification of the “Mediterranean Diet” (MD) by Prof. Ancel Keys is described here, alongside its confirmation through the epidemiological study known as “The Seven Countries Study of Cardiovascular Diseases” (SCS). Prof. Keys’ intuition on the possible dietary determinants of coronary heart disease (CHD) was derived from several pilot studies conducted in various countries. His impression was that the healthy diet was eaten along the Mediterranean shores of Italy, former Yugoslavia, and Greece, characterized by a large intake of bread, cereals, vegetables, fruit, and olive oil, with a small intake of meat, milk, dairy, and sugar products. The SCS was conducted across 16 cohorts of middle-aged men in seven countries (USA, Finland, the Netherlands, Italy, former Yugoslavia, Greece, and Japan), with assessments of usual eating habits, repeated medical examinations, and long-term follow-up. Analyses by Keys on the data from the first 15 years of follow-up indicated that low intake of saturated fatty acids (SAFA), usually derived from animal foods, was associated with the lower occurrence of and mortality from CHD, confirming the idea that a diet such as the Mediterranean Diet could be healthy. Further analyses by collaborators of the SCS, over a longer follow-up period, included the use of food groups and dietary scores of different types, confirming that cohorts with a Mediterranean Diet had a lower risk and death rate from CHD, whereas the reverse occurred in populations consuming an unhealthy diet.

## 1. Preface

In December 2004, just over 20 years ago, Prof. Ancel Keys passed away on the eve of reaching the age of 101 years. Despite the many critics of his scientific work, he has been defined as “a giant in physiology, nutrition, and public health” in a 2021 paper by Prof. JP Montani, University of Fribourg, Switzerland, where an extraordinary summary was described of his various scientific accomplishments [1]. However, Prof. Keys remains primarily associated with the identification of the so-called Mediterranean Diet, which represents a milestone in nutrition science.

This report aims to summarize the story of the Mediterranean Diet as viewed by investigators of the Seven Countries Study of Cardiovascular Diseases (SCS). The data reported here are exclusively derived from previously published material, which are appropriately cited.

## 2. Preliminary Explorations 1950–1957

Prof. Keys was among the first investigators who hypothesized a relationship between dietary habits and chronic diseases, particularly cardiovascular disease. Initially, he was impressed by correlations between FAO (Food and Agriculture Organization) and WHO (World Health Organization) data (for nutrition versus mortality), which suggested a possible relationship of this kind.

In the 1950s, Prof. Keys conducted several pilot studies in different countries, measuring serum cholesterol in small groups of people. They involved experiences in Spain, Greece, Finland, Italy, Japan, and South Africa. The conclusion was that significant differences existed across countries in terms of average serum cholesterol levels. Moreover, meetings with local clinicians, physiologists, and nutritionists in these locations suggested that areas with low serum cholesterol were associated with lower rates of heart attacks, and vice versa. He also observed that populations with low serum cholesterol were characterized by rather simpler eating habits, mainly consisting of large amounts of cereals, vegetables, olive oil, fruits, and small amounts of meat, milk, dairy products, and pastries, a topic on which he carefully inquired.

These characteristics were mainly evident along the shores of Italy, Greece, and former Yugoslavia. Similar patterns came from inspecting the relationship between official eating habits and coronary heart disease (CHD) mortality, derived from FAO and WHO data, respectively, although these findings were considered with much caution.

In 1957, a feasibility study of the SCS was conducted in the rural village of Nicotera in Southern Italy, with the purpose of testing the various procedures of field examinations. Among the collaborators and invited observers, there were potential investigators from several countries [2]. In the end, only a few accepted to join the project, while those who declined the invitation included the French representatives (before the invention of the French paradox) [3].

## 3. Books on Healthy Diet 1959–1975

Two books on healthy diets, the first of which had two editions in 1959 and 1963, were written by Prof. Keys in collaboration with his wife, Ms. Margaret Keys. These books summarized the preliminary explorations in different places and cultures, investigating serum cholesterol levels, local rates of heart attacks, and local eating habits [2,4]. The two books were written in a plain, narrative style, accessible to both professionals and laypeople.

The first book [4] also provided some insights into physiology, medicine, and epidemiology related to the heart, as well as a description of possible risk factors and causes of coronary heart disease (CHD), with an emphasis on serum cholesterol and the possible determinants bound to eating habits. Some notions of nutrition science were also included. The book concluded with a long list of recipes, tested by Ms. Keys, featuring typical Mediterranean dishes.

The second book [2], published in 1975, was subtitled “The Mediterranean Way” and included findings from the first 5 years of the SCS follow-up, published in 1970. It confirmed the hypothesis that was the basis of the study. As a consequence, the first part of the book served as a description of the epidemiological knowledge of CHD, followed by a description of the Mediterranean world, and then the presentation of the results of the SCS, though without numbers or tables. The second part, similar to the first book, consisted of a series of Mediterranean recipes.

Both books were highly successful and became best-sellers. In summary, the Mediterranean Diet identified in these books was characterized by a prevalence of plant foods over animal foods, plus other details. In particular, a relatively large intake of bread and cereals (pasta, rice, and similar foods) should give preference to whole grains products rich in fiber; a large intake of all kinds of vegetables is recommended, including legumes; the same is the case for fruits, possibly including substantial portions of nuts, almonds, and similar items; meat should be consumed with moderation, with an emphasis on excluding red and fatty meats in favor of poultry; fish and seafood should be consumed regularly; milk should be consumed in moderation, with a preference for skim milk and low-fat dairy products; olive oil should be the major dressing and cooking fat, while other hard fats should be excluded; sugar, sugar products, and pastries should be limited to maintain a low glycemic index; and alcohol intake should be moderate, preferable with small amounts of red wine.

## 4. The Seven Countries Study 1958–2024

The Seven Countries Study of Cardiovascular Diseases (SCS) was conceived and started in 1958, enrolling 16 cohorts of middle-aged men (ages 40–59) for a total of 12,763 subjects, in seven countries: USA, Finland, the Netherlands, Italy, former Yugoslavia (in two regions: Croatia and Serbia), Greece, and Japan. These countries were thought to contrast in terms of eating habits. Ten of the cohorts were located in rural communities. Later, another study was launched in Hungary, but it was ultimately not included in the SCS due to a lack of standardization of measurement techniques [2].

The primary goal of the study was to measure various cardiovascular risk factors, focusing on the prevalence, incidence, and mortality rates of CHD over a long follow-up period.

The entry examination included the collection of family and social data, lifestyle behaviors, such as smoking habits and physical activity, a series of anthropometric measurements, a few biochemical and biophysical measurements, diagnoses of prevalent diseases through a complex medical examination, recording of a resting and post-exercise electrocardiogram, and resting spirometry measurements [5]. All measurement techniques underwent strict standardization procedures, and several variables were excluded as they were not suitable for standardization. These included blood glucose, HDL cholesterol, triglycerides, some coagulation indicators, chest X rays, and others. Follow-up consisted of quinquennial field re-examinations (up to year 40 of follow-up, although not regularly carried out across all cohorts), complex procedures to gather additional data on the incidence of major cardiovascular diseases, and collection and coding of mortality data up to year 60 of follow-up in 10 cohorts, after which they were practically extinct. A shorter follow-up period was reached in the remaining six cohorts.

In the early phases of the study, a complex dietary survey was conducted in subsamples of each cohort, including the recording of many food groups and the chemical measurement of some basic nutrients in portions of food taken from the home of the participants [6]. For these operations, significant assistance was provided by renowned nutritionists from several countries, although only a few of them took responsibility for the subsequent operations.

Prof. Keys directed the study with a velvet glove and iron fist, but he is remembered as a kind of patriarch. Many historical details are reported in a dedicated book [7].

## 5. The Seven Countries Study and the Mediterranean Diet Explored by Prof. Keys 1958–1986

The main goal of Prof. Keys, while running and analyzing the SCS, was to show that large differences in saturated fatty acids (SAFA) intake, mediated by mean levels of serum cholesterol, could explain the population differences in CHD incidence and mortality rates. He disregarded the structure of different eating habits, assuming that their coherence with lipid metabolism was given for granted. This approach was partly supported by findings from dietary experiments he conducted in the 1960s, which showed that high intakes of saturated fatty acids (SAFA), usually derived from animal foods, were the major cause of high levels of serum cholesterol [8]. Similar and independent experiments conducted by Hegsted reached the same conclusions [9]. As a consequence, data analyses were initially confined to studying the inter-relationships between SAFA intake, serum cholesterol, and CHD incidence and mortality.

Major findings on this issue were reported, together with other types of analyses, in two monographs and at least two major papers published between 1970 and 1986 [10,11,12,13]. Table 1 summarizes the linear correlation coefficients found at baseline and at 5, 10, and 15 years of follow-up in ecological analyses carried out across the 16 cohorts of the study [10,11,13]. These findings supported Keys’ hypothesis, showing high correlation coefficients between SAFA with serum cholesterol, as well as between SAFA and cholesterol with CHD incidence and mortality during different lengths of follow-up. The last paper in this series was published in 1986 [13], and the predictive role of fatty acids was cumulated with that of other major cardiovascular risk factors in a summary of the long study experience. Moreover, for the first time, the ratio of mono-unsaturated fatty acid/saturated fatty acids (MUFA/SAFA) was tested in prediction models, representing an indirect marker of Mediterranean eating habits. About 30 years later, this paper was selected from the thousands published over the previous 50 years in the American Journal of Epidemiology, classified as a historic contribution, and reprinted in the same journal in 2017, together with 14 other historic papers [14].

Prof. Keys never conducted a proper analysis of the distribution of different food groups across the 16 cohorts. It was only in 1989 that he became a co-author of a paper that eventually provided a careful and comprehensive description of 19 food groups, derived from the dietary surveys conducted at the beginning of the SCS in subsamples from each cohort [15]. However, he was not even a co-author of subsequent papers dealing with the role of these food groups in relation to CHD and other conditions. When Prof. Keys reached his mid-eighties, he became more of a spectator than an active participant in the subsequent operations of the SCS.

After this historic paper, Prof. Keys published only one more brief review on the study in 1995, when he was already 91 years old. In this review, he used the term “Mediterranean Diet” in the title, summarizing his experiences and offering some personal reflections [16].

## 6. The Seven Countries Study and the Mediterranean Diet Explored by Collaborators of the SCS 1987–2025

After Prof. Keys’ retirement from active participation in the SCS, the responsibility for further analyses of the Mediterranean Diet shifted to a group of investigators belonging to the first, second, and third generations of the SCS.

The first paper examining the relationships between food groups and CHD mortality was published in 1999 [17], where two complex, a-posteriori dietary scores were derived from the 19 food groups across the 16 cohorts. The ecological associations with 25-year CHD mortality, expressed by linear correlation coefficients, were 0.89 and 0.87 (highly significant). Low levels of these scores were associated with a dietary profile corresponding to that of the typical Mediterranean Diet, providing evidence for the protective role of this diet.

Following some preliminary tests conducted on local data [18,19], a group of Italian SCS investigators proposed an easier-to-use a-priori dietary score called MAI (Mediterranean Adequacy Index). It exploited data from the dietary survey conducted in Nicotera, a rural village in Southern Italy, where a feasibility survey of the SCS was conducted in 1957 [20,21]. It is based on the ratio of food groups (expressed as percent of total energy) from vegetable sources and fish, over food groups from animal sources and sugar products. The first group includes bread, cereals, legumes, potatoes, vegetables, fruits, oils, fish, and wine in the numerator, while milk, cheese, meat, eggs, animal fat, margarine, sugars, cakes, sugar products, and sweet beverages were the components of the denominator. High values of the MAI correspond to the structure of a “typical” Mediterranean Diet. The natural logarithm of MAI (lnMAI) was found to be highly and inversely associated with 25-year mortality from CHD in the 16 cohorts of the SCS [20,21].

Two major ecological analyses of the SCS involved the prediction of 50-year CHD and all-cause mortality as a function of various food groups, some nutrients, and mainly the lnMAI [22,23]. On this occasion, some slight changes were adopted to the levels of nutrients following a replica of the chemical measurements made on representative food groups from each cohort [24]. High levels of lnMAI were associated with a significantly lower risk of CHD mortality, with a linear correlation coefficient of −0.91. The corresponding linear partial correlation coefficient for all-cause mortality was 0.62 (significant), after adjusting for socio-economic status.

In two subsequent papers, the association between lnMAI and other cardiovascular mortality endpoints, such as HDUE (heart diseases of uncertain etiology) and stroke [25], was studied. The analysis was extended to 60 years of follow-up for 10 out of the 16 cohorts, after which they reached extinction [26]. In this case, the strong negative association between lnMAI and CHD mortality was confirmed, while the two alternative CVD groups were directly associated with lnMAI, suggesting that they are different diseases compared to CHD or that their relationship with lipid metabolism is different. Part of this problem was explained by an analysis of competing risks, comparing CHD events with HDUE and stroke, which showed the critical role of serum cholesterol in segregating these two conditions from CHD [27]. In fact, there were positive relationships between serum cholesterol and CHD mortality, and negative relationships between HDUE and stroke deaths.

A summary of the major associations between lnMAI and long-term cardiovascular mortality is provided in Table 2. In these more recent analyses, lnMAI was identified as the first of four coherently connected steps linking diet with CHD mortality through its association with dietary atherogenicity and thrombogenicity indexes, as well as serum cholesterol. This approach provided an almost complete picture of key dietary and metabolic determinants of CHD events [25,26].

Around three decades after the initial examination, new dietary surveys were repeated among the survivors from some of the areas, including those where the basic dietary habits were “Mediterranean”, such as Greece and Italy. A reduced intake of typical Mediterranean food groups was documented in both countries, together with an increase in some non-Mediterranean components [28]. This was particularly true in Greece, where it was associated with a significant increase in average serum cholesterol levels and an acceleration of CHD mortality rates. On the other hand, although less relevant, inverse changes were observed in Finland and the Netherlands. However, these changes were insufficient to modify the long-term overall CHD mortality rankings across the various cohorts [29].

## 7. Seven Countries Conclusions 2025

The main goal of the SCS was to provide solid evidence for the hypotheses proposed by Prof. Keys in his books about the Mediterranean Diet. The study largely confirmed his ideas about the relationship between SAFA, serum cholesterol, and the incidence and mortality of CHD.

However, some aspects related to the food groups of the Mediterranean Diet remained unresolved. Despite the valuable dietary surveys carried out at the beginning of the study, the definition of a common set of food groups valid and homogeneous across all the 16 cohorts was limited to 19 groups, such as bread, cereals, potatoes, vegetables, legumes, fruit, oils, meat, fish, eggs, butter, other hard fats, milk, cheese, pastries, sugar, sugar products, alcohol, and sweet beverages. As a consequence, despite Prof. Keys’ intuitions expressed in his diet books, some important details were missing, such as the distinction between bread and cereals rich or poor in fiber, fresh versus dry fruit (like nuts and similar items), red meat versus other types of meat (like poultry), fat fish versus lean fish, whole milk versus skim milk, fat versus lean dairy products, and olive oils versus other oils. This situation may have had consequences on part of the analyses carried out subsequently by the collaborators of Prof. Keys regarding food groups.

On the other hand, Prof. Keys’ concerns about SAFA were maintained in later analyses, particularly those examining the details of various fatty acids. Additionally, the long-term follow-up provided further valuable insights.

## 8. Other Studies 1980–2020

In the early 1980s, an independent experiment provided large support to the ideas of Prof. Keys linking dietary habits changes with disease. The study investigated, in small population groups, the relationship between dietary habits and serum cholesterol and was composed of two parallel investigations. One was carried out in a Finnish community where the diet was changed toward the Mediterranean style [30], while the other, conducted in southern Italy, shifted the Mediterranean Diet toward a high saturated fat intake, typical of the usual Finnish diet [31]. Findings showed a substantial decrease in serum cholesterol levels in Finland and a significant increase in Italy.

Incidentally, we have the impression that in the 1984 paper by Ferro-Luzzi et al. [31], the term “Mediterranean Diet” was probably used for the first time in the title of a paper and was frequently repeated in the text. While we cannot be fully sure about this fact, it is clear that later on, the term became rather common in scientific papers and lay media.

Around the early 1990s, other investigators, mainly from the USA, Spain, and Greece, started to expand the study of the Mediterranean Diet in other populations and cultures, providing a large contribution to the topic. An important stimulus was given by the creation and publication of the so-called Mediterranean Diet Pyramid, which summarized in a simple figure the structure of ideal Mediterranean eating habits [32]. A fundamental contribution came from the Spanish preventive trial of the Mediterranean Diet carried out by Estruch and collaborators, where the intervention group’s diet was complemented by extra doses of olive oil or nuts and similar dried fruits [33]. The final findings showed an impressive reduction in CHD events in the treatment group versus the control group, exploiting only two of the specific components of the Mediterranean Diet.

The interest in the Mediterranean Diet spread widely in research activities, and so far, the bibliographic PUBMED platform has reported more than 12,000 contributions [34]. Moreover, we have identified at least three books dedicated to the topic [35,36,37]. In one of these books, two investigators from the SCS published a chapter that critically reviewed the MAI [38]. Simply considering the content of these three books, it appears that a large number of endpoints were considered to be possibly related to the Mediterranean Diet, and that almost all analyses indicated a significant protective role of the diet. All this is true even though the dietary scores representing the Mediterranean Diet were created in many different fashions. In fact, the Mediterranean Diet derived from Prof. Keys’ books and SCS analyses, which prioritized the preference for vegetable foods over animal foods, served as the basis for further use, though new additions and changes were introduced. In most of those scores, such as the HEI (Healthy Eating Index) [39] and the mHEI (metric Healthy Eating Index) [40], there was an insistence in the choice of carbohydrate foods rich in fiber, low-fat or skim milk and dairy products, the preference for olive oil as the primary fat, and the limitation of sugar and sugar products. All of this aligned with the description of the Mediterranean Diet in Prof Keys’ books, details that could not be considered in the SCS 

## 9. Consequences and Recognitions

In 2010, the Mediterranean Diet was recognized by UNESCO as an Intangible Heritage of Humanity. Beyond its role in physiology, medicine, nutrition science, and prevention, other aspects were recognized, such as the interplay of skills, knowledge, processing, cooking, and the sharing and consumption of food.

These socio-cultural aspects were further evaluated by other initiatives, such as the one of the S. Orsola Benincasa University of Naples, which established a Center for Social Research and a Virtual Museum on the Mediterranean Diet.

## 10. Conclusions

Beyond the infinite debate about the role of dietary SAFA in the origin of atherosclerosis and CHD [3], the identification of the Mediterranean Diet, which is low in dietary SAFA, appears to be a significant scientific accomplishment and a valuable tool for maintaining health, preventing disease, and promoting longevity [38,39,40,41]. Despite some variations in its practical interpretation, it remains a solid basis for overall good health in the absence, at the moment, of any valuable alternative.

## Figures and Tables

**Table 1:** Ecological correlations of SAFA, serum cholesterol, and CHD incidence and mortality at baseline, and at 5, 10, and 15 years of follow-up. Data from references [10,11,13].

Correlated Variables	R	R^2^	*p* Value
**Baseline**			
SAFA versus Cholesterol	+0.89	0.79	<0.0001
SAFA versus Cholesterol *	+0.90	0.81	<0.0001
**5-year follow-up**			
SAFA versus CHD incidence	+0.84	0.71	<0.0001
Cholesterol versus CHD incidence	+0.76	0.58	0.0007
**10-year follow-up**			
SAFA versus CHD incidence	+0.73	0.53	0.0013
SAFA versus CHD mortality	+0.84	0.71	<0.0001
Cholesterol versusCHD mortality	+0.80	0.64	<0.0001
**15-year follow-up**			
SAFA versus CHD mortality	+0.89	0.79	<0.0001
MUFA/SAFA versus CHD mortality	+0.66	0.44	0.0054
MUFA/SAFA ** versus CHD mortality	+0.98	0.96	<0.0001

CHD: coronary heart disease; SAFA: dietary saturated fatty acids; MUFA: dietary mono-unsaturated fatty acids. * Serum cholesterol estimated by the Keys equation [8]. ** Together with age, body mass index, cigarettes per day, systolic blood pressure, and serum cholesterol. R = linear correlation coefficient; R^2^ = proportion of variability explained by R.

**Table 2:** Ecological correlations of lnMAI with mortality from CHD, HDUE, and stroke and year 25 of follow-up (16 cohorts), year 50 of follow-up (16 cohorts), and year 60 of follow-up (10 cohorts). Data from references [20,23,25,26].

Correlated Variables	R	R^2^	*p* Value
lnMAI versus CHD mortality 25 years	−0.84	0.71	<0.0001
lnMAI versus CHD morality 50 years	−0.91	0.83	<0.0001
lnMAI versus CHD mortality 60 years	−0.98	0.96	<0.0001
lnMAI versus HDUE mortality 50 years	+0.01	0.0001	0.9706
lnMAI versus HDUE mortality 60 years	+0.23	0.05	0.3914
lnMAI versus Stroke mortality 50 years	+0.42	0.18	0.1054
lnMAI versus Stroke mortality 60 years	+0.96	0.92	<0.0001

lnMAI: natural logarithm of Mediterranean Adequacy Index; CHD: coronary heart disease; HDUE: other heart diseases of uncertain etiology; Stroke: cerebrovascular diseases. R = linear correlation coefficient; R^2^ = proportion of variability explained by R.

## Data Availability

The authors presented only data published in the reported references, properly cited, and did not use original data from the Seven Countries Study’s original files.
